# Differential Network-Based Dietary Structure and Type 2 Diabetes Risk: A Prospective Cohort Study Using Food Co-Consumption Networks

**DOI:** 10.3390/nu18030506

**Published:** 2026-02-02

**Authors:** Hye Won Woo, Yu-Mi Kim, Min-Ho Shin, Sang Baek Koh, Hyeon Chang Kim, Mi Kyung Kim

**Affiliations:** 1 Department of Preventive Medicine, College of Medicine, Hanyang University, Seoul 04763, Republic of Korea; woohehe@hanyang.ac.kr (H.W.W.); kimyumi@hanyang.ac.kr (Y.-M.K.); 2 Institute for Health and Society, Hanyang University, Seoul 04763, Republic of Korea; 3 Department of Preventive Medicine, Chonnam National University Medical School, 160 Baekseo-ro, Dong-gu, Gwangju 61469, Republic of Korea; mhshinx@naver.com; 4 Department of Preventive Medicine, Yonsei University Wonju College of Medicine, 20 Ilsan-ro, Wonju 26426, Republic of Korea; kohhj@yonsei.ac.kr; 5 Department of Preventive Medicine, Yonsei University College of Medicine, 50 Yonsei-ro, Seodaemun-gu, Seoul 03722, Republic of Korea; hckim@yuhs.ac

**Keywords:** co-consumption, differential networks, Type 2 diabetes, cross-cohort validation, Korea

## Abstract

**Background/Objectives**: Current data-driven dietary pattern methods have limitations in identifying disease-specific dietary structures. We developed network-derived dietary scores based on type 2 diabetes (T2D)-differential food co-consumption networks and examined their associations with incident T2D risk. **Methods**: Using the Korean Genome and Epidemiology Study-CArdioVascular disease Association Study (KoGES-CAVAS, *n* = 16,665), we constructed food co-consumption networks from cumulative average intakes stratified by incident T2D status. The network centrality scores from edges appearing exclusively in either T2D or non-T2D networks were used to generate a differential co-consumption network-derived (D_CCN) score, with higher scores indicating a greater alignment with diabetes-specific structures. CAVAS-derived scores were applied to the Health Examinee Study (KoGES-HEXA, *n* = 51,206) for cross-cohort validation. Incidence rate ratios (IRRs) were estimated using modified Poisson regression with robust error estimation. **Results**: During follow-up, 953 and 2190 new cases of T2D were identified in CAVAS and HEXA, respectively. Rice and vegetable dishes were primary hub foods in both networks, with rice showing exclusively negative correlations. Non-T2D networks were more complex, whereas T2D networks were simpler and centered on refined flour-based foods. The D_CCN score was associated with a higher T2D risk in CAVAS (IRR = 1.45, 95% CI: 1.21–1.74), and this association was validated in HEXA (IRR = 1.58, 95% CI: 1.40–1.78), with consistent dose–response relationships (both *p*-trend < 0.0001). **Conclusions**: Differential network analysis identified T2D-specific co-consumption structures, and the D_CCN score consistently predicted T2D risk across cohorts. This approach highlights the utility of network-based methods for capturing disease-relevant dietary structures beyond traditional approaches.

## 1. Introduction

Dietary patterns are strongly associated with type 2 diabetes (T2D) risk, with healthy dietary patterns (e.g., Mediterranean diet and plant-based diets) linked to a 13–19% reduction and unhealthy patterns (e.g., high in red/processed meat and refined grains) linked to a 44% increase in risk [1,2]. However, identifying dietary patterns that are specifically related to disease development remains challenging. This is partly because widely used analytical approaches such as factor analysis were not designed to capture disease-dependent differences in dietary structure [3,4].

Current dietary pattern methods face three major limitations. First, traditional hypothesis-driven (a priori) scores rely on fixed criteria that may overlook culturally specific eating behaviors [5]. Second, exploratory (a posteriori) approaches derive dietary patterns from food–food correlations estimated within the study population. As a result, the identified patterns reflect general eating behaviors rather than disease-specific structures, and the patterns derived in one study population may not correspond to risk-relevant patterns in another population [6,7]. Third, although novel approaches such as network analysis have emerged, models like Gaussian Graphical Models assume multivariate normality and perform poorly with the zero-inflated and skewed distributions typical of dietary data [8,9,10]. In addition to these methodological limitations, population-level characteristics—such as the relatively homogeneous dietary habits observed in some Asian populations [11,12]—reduce dietary variability, making it more difficult to distinguish dietary pattern subtypes and detect their associations with disease [13].

Food co-consumption network (CCN) analysis may provide a way to overcome these methodological limitations by capturing structural differences in how foods are consumed together by disease status [14,15,16]. Unlike traditional approaches such as PCA or factor analysis, which summarize the overall covariance among foods into a limited number of latent patterns, CCN analysis preserves the explicit food-to-food relationship structure by modeling pairwise co-consumption links [14]. This allows CCNs to capture differences in how foods are consumed together—structural features that are not retained in conventional pattern-reduction methods. Although reduced rank regression (RRR) incorporates disease-related response variables [7], it likewise does not preserve pairwise co-consumption relationships or group-specific network topology. In contrast, differential network analysis directly compares co-consumption structures between individuals who develop disease and those who do not, yielding disease-specific network signatures [14]. Because individuals may consume similar sets of foods but differ in how those foods are combined, co-consumption edge structures (pairwise co-consumption relationships between foods) can vary across disease status, allowing CCN-based approaches to detect structural differences even when the overall dietary variability is limited.

Therefore, this study aimed to develop a differential co-consumption network-derived (D_CCN) score based on T2D-specific co-consumption structures in the KoGES-CAVAS cohort and examine its association with incident T2D, then apply the score to the KoGES-HEXA cohort to evaluate external validity.

## 2. Materials and Methods

### 2.1. Study Design and Population

This study used data from two population-based cohorts from the Korean Genome and Epidemiology Study (KoGES): the CArdioVascular disease Association Study (CAVAS) [17] and the Health Examinee (HEXA) Study [18].

CAVAS is a prospective community-based cohort established in 2005 to investigate cardiometabolic risk factors among adults aged ≥ 40 years residing in six rural regions of Korea (Yangpyeong, Namwon, Goryeong, Wonju, Pyeongchang, and Kangwha). Between 2005 and 2011, 19,545 participants without cardiovascular disease or cancer were enrolled and followed up with every 2–4 years through 2017, with 78.2% completing at least one follow-up examination [17]. HEXA is a large-scale community-based cohort conducted primarily in urban areas to investigate chronic disease determinants. Between 2004 and 2013, 173,195 adults aged 40–79 years were recruited from 38 hospitals and health examination centers nationwide; 161,053 participants without cardiovascular disease or cancer were eligible at baseline, and 65,608 completed a follow-up examination between 2012 and 2016 (median interval 4.25 years) [18].

For both cohorts, participants were excluded if they had prevalent diabetes at baseline (use of anti-diabetic medication or insulin, or fasting blood glucose (FBG) ≥ 126 mg/dL), missing or implausible dietary data (≥99.5th or ≤0.5th percentile of energy intake), or missing key covariates [education, exercise, smoking, alcohol or body mass index (BMI)]. After exclusions, 16,665 participants from CAVAS (6162 men and 10,503 women) and 51,206 participants from HEXA (16,536 men and 34,670 women) were included in the final analysis. Details of participant selection are shown in Supplementary Figure S1.

All participants provided written informed consent, and the study was approved by relevant Institutional Review Boards in accordance with the Declaration of Helsinki.

### 2.2. Dietary Assessment

Dietary intake was assessed at baseline, and during follow-up surveys administered by trained interviewers using the same validated 106-item Food Frequency Questionnaire (FFQ) in both cohorts. The FFQ includes 106 food items with nine frequency categories, ranging from “never or rarely” to “three times/day”, and specifies three portion sizes for each item [19]. Daily food consumption (servings/day) was calculated by multiplying the weighted frequency by the average serving size for each food. To determine optimal food groupings, the 106 food items were initially aggregated into 47 food groups, as described in a previous study [20]. In the present study, we further combined three rice-related groups (cooked white rice, cooked rice with beans, and cooked rice with multi-grains) into a single rice category, resulting in a total of 45 food groups (hereafter referred to as foods) (Supplementary Table S1). Energy intake was calculated using the nutrient database developed by the Korean Nutrition Society, based on the seventh edition of the Korean Food Composition Table [21]. To account for long-term dietary patterns and reduce measurement error [22], cumulative average intakes of the 45 food groups and total energy were calculated by averaging dietary assessments collected prior to disease diagnosis or censoring. In CAVAS, T2D status was assessed at each visit, and dietary intake was assessed at baseline and at each follow-up visit (up to three dietary assessments per participant); all dietary assessments prior to T2D diagnosis or censoring were averaged. In HEXA, T2D status was assessed at each visit, and dietary intake was assessed at baseline and at one follow-up visit; if T2D was diagnosed before follow-up, only baseline dietary data were used; otherwise, both assessments were averaged.

### 2.3. Ascertainment of Diabetes

At each visit, participants were asked whether they had been diagnosed with T2D by a physician and whether they were being treated with anti-diabetic drugs and/or insulin. A diagnosis of diabetes was defined as meeting any of the following criteria, as recommended for FBG by the American Diabetes Association (ADA) [23]: (1) FBG ≥ 126 mg/dL (7.0 mmol/L) or (2) current treatment with oral hypoglycemic agents or insulin.

### 2.4. Assessment of Covariates

Data on age, educational level, and risk factors for T2D (regular exercise, smoking status, alcohol consumption, drinking status, and BMI) were collected using standardized interview-based questionnaires and health examinations. All examinations were conducted according to standardized protocols to ensure consistency across study centers. Height was measured to the nearest 0.1 cm with a stadiometer, and weight to the nearest 0.1 kg using a calibrated scale, with participants wearing light clothing and no shoes. BMI was calculated as weight in kilograms divided by height in meters squared (kg/m^2^). Blood samples were collected after an overnight fast of at least 8 h, and serum FBG concentrations were determined using an ADVIA 1650 Automated Analyzer (Siemens, New York, NY, USA) in both the CAVAS and HEXA cohorts.

### 2.5. Statistical Analysis

Data for the general characteristics across both cohorts were summarized as means ± standard deviation (SD) for continuous variables and percentages for categorical variables.

Co-consumption network (CCN) analysis was performed based on a previously proposed network approach [14] (see also Supplementary Figure S2). In the first stage of the food-CCN analysis, edge weights (i.e., links between foods), representing the strength of food–food co-consumption, were calculated based on the partial Spearman correlations among 45 food groups using continuous intake (servings/day). These correlations were adjusted for total energy intake and sex, which are key confounders of both food consumption patterns and T2D risk [24,25,26]. To ensure that the network captured only stable and meaningful food–food relationships, the dataset was randomly subsampled by 50%, and this procedure was repeated 100 times. Edges were retained only if the average correlation coefficient across iterations exceeded 0.2 and the correlation was statistically significant (*p* < 0.05) in all 100 iterations. This threshold (Spearman’s rank correlation |r| > 0.2) corresponded to correlation strengths above the 75th percentile (Q3) of the overall correlation distribution in the CAVAS (75th percentile r = 0.166) (Supplementary Figure S3).

In the second stage of the food-CCN analysis, separate food co-consumption networks were constructed for participants who developed T2D and for those who remained diabetes-free. We define the differential co-consumption network as a network representing dietary structural differences between individuals who develop T2D and those who do not, constructed by identifying links (food–food relationships) that are exclusive to either the non-diabetic or diabetic network (Supplementary Figure S4). The rationale for this approach is that the diabetes and non-diabetes networks in populations with relatively homogeneous dietary practices share a substantial structural overlap, reflecting a common dietary background; by excluding shared edges, the differential network isolates co-consumption relationships exclusive to each disease status, enhancing disease-specific signals. This resulted in two group-specific differential subnetworks: diabetes-specific and non-diabetes-specific. For each subnetwork, five centrality measures—degree, betweenness, closeness, eigenvector, and strength—were calculated to quantify the structural importance of each food within the network, capturing complementary aspects of food importance, including connections (degree), interaction strength (strength), position (closeness), mediation (betweenness), and influence (eigenvector), while maintaining interpretability and parsimony. The five centrality measures were standardized using z-scores and then averaged to obtain an integrated centrality value.

Individual network-based dietary scores were derived by weighting each participant’s binarized intake (0/1) by the corresponding integrated centrality values in each subnetwork. To determine consumption status, binarization was applied because the primary aim of this study was to capture co-consumption relationships—whether foods are consumed together—rather than quantify absolute intake levels. Because dietary intake data typically contain many zeros (indicating non-consumption) and are highly skewed, each food group was first converted into binary form using adaptive cut-points derived from its own distribution. Specifically, cut-points were determined in a hierarchical manner to reflect the consumption pattern of each food: (1) if the first quartile (Q1) was non-zero, it was used as the cut-point; (2) if Q1 was zero but the median was greater than zero, the median was used; (3) if both Q1 and the median were zero, the third quartile (Q3) was used; and, (4) for foods where all quartiles (Q1, the median, Q3) were zero, any amount above zero was considered as consumption. Thus, values above the cut-point for each food were coded as 1 (consumed), and those below the cut-point were coded as 0 (not consumed).

The differential co-consumption network-derived (D_CCN) score was defined as the difference between the two network-based dietary scores (Diabetes Score–Non-diabetes Score), representing the extent to which an individual’s diet aligns with the T2D-associated co-consumption structure. Unlike traditional dietary pattern scores that measure adherence to predefined food compositions, the D_CCN score captures structural alignment with disease-specific food networks. To help illustrate which foods emerged as top-ranking foods in the diabetes-specific and non-diabetes-specific subnetworks, and how broadly each of these foods was connected with other foods, we constructed 2-step ego networks centered on key hub foods. A 2-step ego network visualizes a focal food together with the foods that are commonly consumed with it, as well as additional foods that are indirectly connected through those combinations, allowing for a broader view of how the focal food is embedded within the overall dietary structure. Finally, for external validation, the integrated centrality values derived from the CAVAS differential co-consumption networks were used to compute individual D_CCN scores in the HEXA cohort by weighting each participant’s food intake with the corresponding CAVAS-derived weights.

Incidence rate ratios (IRRs) for T2D were calculated using modified Poisson regression models with robust error estimation and log-transformed person-time as an offset term [27,28]. In both cohorts, participants were categorized into quartiles based on the distribution of D_CCN scores within each cohort, and IRRs were calculated for each quartile relative to the lowest quartile as a reference. Covariates including age (years), sex, higher education level (≥12 years), regular exercise (≥3 times/week for ≥30 min/session), current smoking status, alcohol consumption (g/day), BMI (kg/m^2^), and total energy intake (kcal/day) were adjusted in the models. All network analyses were conducted in R statistical software (version 4.3.0) using the igraph package for network construction and centrality calculations. Statistical analyses for IRR estimation were performed using SAS software (version 9.4; SAS Institute Inc., Cary, NC, USA).

## 3. Results

The study participants included 16,665 participants from CAVAS (mean age 58.2 ± 9.7 years; 63.0% women) and 51,206 participants from HEXA (mean age 53.0 ± 7.9 years; 67.7% women). Over follow-up periods of 5.8 ± 3.9 years (CAVAS) and 4.9 ± 1.8 years (HEXA), 953 and 2190 participants developed T2D, corresponding to incidence rates of 9.9 and 8.7 per 1000 person-years, respectively (Table 1). In both cohorts, participants who developed T2D were more likely to be male, older, or current smokers, and had a higher BMI, whereas educational attainment was lower among T2D cases. Regular exercise showed minimal differences.

**Figure 1:**
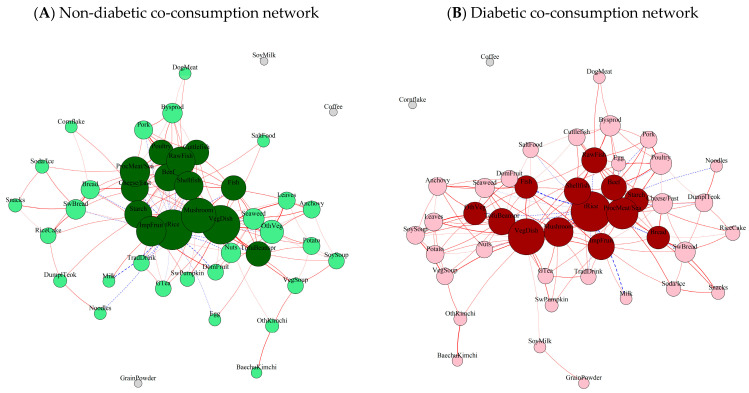
Food co-consumption networks based on sex and energy-adjusted partial correlations in participants who remained diabetes-free (**A**) and those who developed diabetes (**B**) in CAVAS. Nodes represent foods, with size proportional to degree centrality. Node colors distinguish connectivity levels in non-diabetic network (dark green for high-degree hub foods, light green for regularly connected foods) and diabetic network (dark red for high-degree hub foods, light pink for regularly connected foods); gray nodes represent isolated foods with no significant connections. Edges represent consistently significant partial correlations (100-iteration average r > 0.2, *p* < 0.05 in all iterations) adjusted for sex and total energy intake, with red edges indicating positive correlations and blue edges indicating negative correlations between food groups. Edge width is proportional to the absolute value of partial correlation coefficients, representing the strength of co-consumption relationships.

Figure 1 shows diabetes-specific and non-diabetes-specific food co-consumption networks based on the 45 predefined food groups. The non-diabetes network comprised 177 edges and the diabetes network comprised 175 edges, indicating broadly similar network structures (Supplementary Table S2). Sensitivity analyses showed that the network structures remained stable across alternative correlation thresholds (|r| > 0.15, 0.25, 0.30) and bootstrap iterations (50, 75, 100), with 99.4–100% of edges achieving significance across all iterations. Total rice was the most prominent hub in both networks, exhibiting the highest degree centrality (24 connections in the non-diabetes network, 25 in the diabetes network), followed by vegetable dishes (23 vs. 22 connections), mushrooms (20 vs. 16 connections), and processed meat/seafood (16 vs. 18 connections). Centrality rankings for the top 10 hub foods are presented in Table 2. Rice was involved in all negative correlations (blue dashed edges) in both networks. Several foods appeared as isolated nodes, including grain powder, soy milk, and coffee in the non-diabetes network, and cornflakes and coffee in the diabetes network.

**Figure 2:**
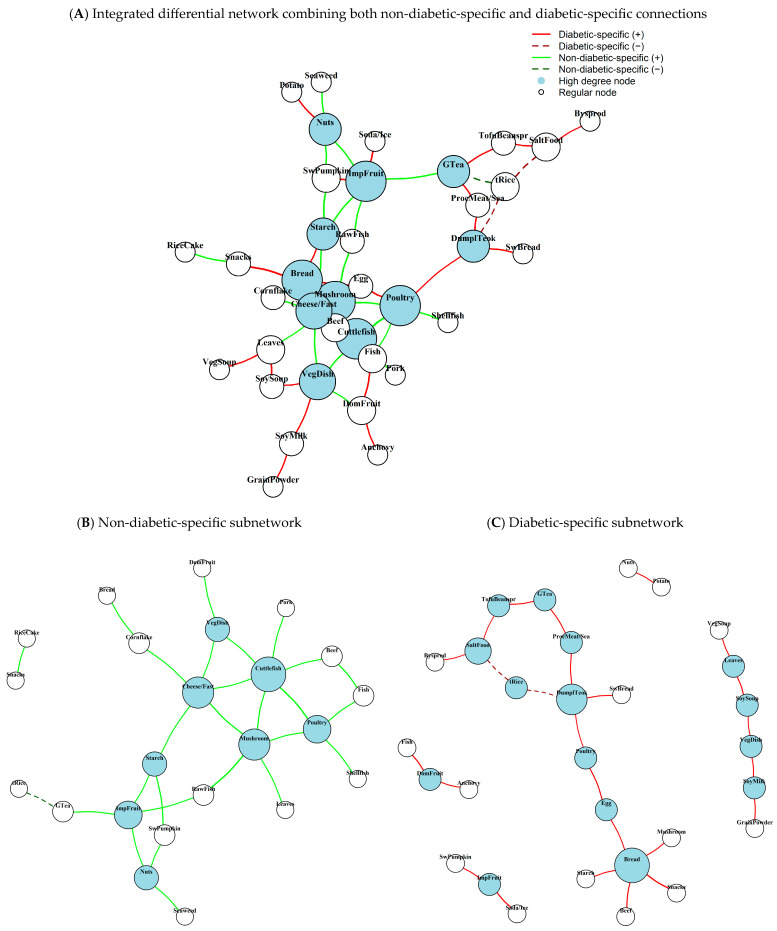
**Differential co-consumption networks by diabetes status in CAVAS cohort.** (**A**) Integrated differential network combining both non-diabetic-specific (green edges) and diabetic-specific (red edges) connections. (**B**) Non-diabetic-specific network showing food–food connections appearing exclusively in participants who remained diabetes-free. (**C**) Diabetic-specific network displaying connections appearing exclusively in participants who developed diabetes. Node size is proportional to degree centrality within each respective network. Blue nodes indicate high-degree hub foods (≥75th percentile of degree distribution), while white nodes represent regularly connected foods. Solid edges indicate positive partial correlations, and dashed edges indicate negative correlations. All edges represent consistently significant partial correlations (100-iteration average r > 0.2, *p* < 0.05 in all iterations) adjusted for sex and total energy intake.

Differential subnetworks highlighting the structural differences between the diabetes and non-diabetes groups are shown in Figure 2 (combined visualization in Figure 2A; group-specific subnetworks in Figure 2B,C). The non-diabetic differential network contained 23 food nodes with 27 edges, whereas the diabetic differential network included 29 food nodes with 25 edges. Distinct hub foods were identified in each subnetwork based on integrated centrality values (Table 3). In the non-diabetic differential network, cuttlefish emerged as the primary hub food (integrated centrality value = 3.510), followed by cheese and pizza/hamburgers (3.423) and mushrooms (3.323). In contrast, the diabetic differential subnetwork was characterized by breads/spreads as the primary hub (4.073), followed by dumplings and rice cake soup (*Tteokguk*) (3.319) and eggs (2.496).

**Figure 3:**
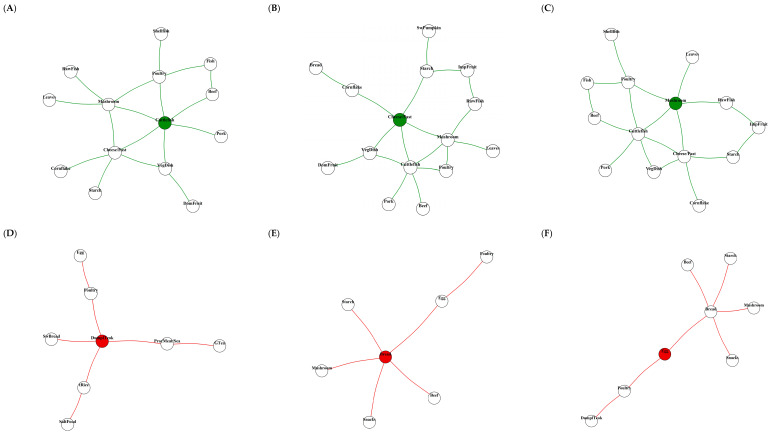
Local neighborhood structures of hub foods in diabetes-specific and non-diabetes specific subnetworks in CAVAS cohort. 2-step ego networks showing local co-consumption structures of central foods in subnetworks. (**A**–**C**) In the non-diabetes-specific subnetworks: cuttlefish, cheese and pizza/hamburger, and mushroom networks. (**D**–**F**) Diabetes-specific structures: bread/spread, dumpling and *Tteokguk*, and egg networks. Each network displays the focal food (colored node) and its directly connected neighbors within the differential co-consumption structure.

Ego network visualizations are presented in Figure 3. Non-diabetes-specific ego networks (Figure 3A–C) showed more complex and interconnected structures, with larger networks centered on cuttlefish, cheese and pizza/hamburgers, and mushrooms (mean: 14.3 nodes, 17.7 edges), a higher average degree (2.47), and evident peripheral clustering (clustering coefficient 0.196). In contrast, the diabetes-specific ego networks (Figure 3D–F) exhibited simpler, hub-centered structures with a smaller network size (mean 7.67 nodes, 6.67 edges), lower average degree (1.74), higher density (0.262), and little evidence of peripheral clustering.

Table 4 shows the associations between quartiles of D_CCN scores and T2D incidence in both cohorts. In the CAVAS cohort, D_CCN scores ranged from −9.90 to 24.6. A higher D_CCN score was positively associated with T2D risk in the multivariable model (IRR = 1.45 in Q4 vs. Q1, 95% CI: 1.21–1.74, *p* for trend <0.0001). This association remained robust after additional adjustment for baseline fasting blood glucose (IRR = 1.39, 95% CI: 1.16–1.67, *p* for trend = 0.0001). In the external validation cohort (HEXA), a similar positive association was observed, with participants in Q4 exhibiting a 58% higher risk of incident T2D (IRR = 1.58, 95% CI: 1.40–1.78, *p* for trend <0.0001 in multivariable model).

## 4. Discussion

Using a differential co-consumption network (D_CCN) approach, we identified distinct food co-consumption structures associated with incident T2D. The resulting network-based dietary score showed consistent associations with T2D risk in both the CAVAS and HEXA cohorts. Across both diabetes-specific and non-diabetes-specific networks, rice and vegetable dishes consistently emerged as primary hub foods, underscoring their central roles in the Korean diet. Differential network analysis further showed that the non-diabetes-specific network exhibited more diverse and interconnected food co-consumption structures, whereas the diabetes-specific network was characterized by more simplified, hub-centered structures dominated by refined flour-based foods such as breads/spreads and dumplings/*Tteokguk*. Importantly, the D_CCN scores derived from the CAVAS cohort were consistently associated with incident T2D risk in the independent HEXA cohort, supporting the external validity and cross-cohort transferability of this approach.

A key methodological novelty of this study lies in extending co-consumption network analysis beyond descriptive, group-level characterization to individual-level risk quantification. Previous CCN-based studies have demonstrated that how foods are consumed together—rather than only how much of each food is consumed—differs between disease and non-disease groups and may be relevant for disease development [14,15,29]. However, most existing approaches have remained largely descriptive, focusing on visualizing or comparing network structures, and have lacked a framework for translating these structural differences into scalable, individual-level scores. This limitation is particularly evident in populations with relatively homogeneous dietary practices, such as Korean diets centered on rice and vegetables [30], where conventional module-based network approaches and unsupervised dietary pattern methods often fail to capture disease-relevant structures. Prior methodological studies in T2D have similarly shown that outcome-informed approaches yield stronger associations than patterns derived independently of disease status [31,32], highlighting the need for disease-specific network frameworks that enable individual-level risk quantification. To address these methodological gaps, this study proposes a differential co-consumption network framework that incorporates disease status into network construction and leverages centrality-based features to translate group-level dietary structures into individual-level risk characterization. In this context, the proposed framework can be viewed as a methodological extension of prior CCN approaches, tailored to disease-specific dietary risk assessment. To empirically test whether D_CCN captures distinct dietary information, we examined its correlations with established dietary pattern scores and compared their predictive performance. D_CCN scores showed weak correlations with established dietary pattern scores, including the Mediterranean diet score (MDS), Dietary Approaches to Stop Hypertension score (DASH), and plant-based diet index (PDI) (r = 0.08–0.25), indicating that D_CCN captures dietary characteristics that are largely independent of traditional intake-based measures. Notably, the weak positive correlation with PDI (r = 0.25) reflects a limitation of the overall PDI, which assigns positive scores to all plant foods without distinguishing between healthy (e.g., whole grains, fruits) and less healthy (e.g., refined grains) options. In Korean diets, where rice is a dietary staple, a high rice consumption contributes positively to PDI while also characterizing diabetes-associated co-consumption structures captured by D_CCN. Despite their weak positive correlation, PDI and D_CCN predicted T2D risk in opposite directions: PDI showed a protective association (IRR = 0.64), whereas D_CCN showed an adverse association (IRR = 1.39). After mutual adjustment, the protective effect of PDI strengthened (IRR: 0.64 → 0.59), and, conversely, the adverse effect of D_CCN also strengthened (IRR: 1.39 → 1.52). This suggests that individuals with a higher plant-based food intake may nonetheless exhibit diabetes-associated co-consumption structures. These findings support that dietary quality—what and how much individuals eat—and dietary structure—how foods are combined—operate as complementary predictors of T2D risk.

A notable finding in this network analysis was the consistent emergence of rice and vegetable dishes as the primary hub foods across both diabetic and non-diabetic networks. Rice demonstrated the highest degree centrality (24 connections in the non-diabetic network, 25 in the diabetic network), followed by vegetable dishes (23 and 22 connections, respectively). This central positioning reflects their fundamental roles in Korea’s traditional meal structure, where rice historically served as the main staple and energy source, while vegetable dishes (*banchan*) functioned as essential accompaniments [33]. Interestingly, rice showed a unique network behavior: although it served as the core hub food, it was involved exclusively in negative correlations with other foods. This pattern suggests that a higher rice consumption is associated with a lower consumption of other food groups, indicating a substitution-like role rather than co-consumption. Such a structural feature may reflect a limited dietary diversity within meals, highlighting how network-based approaches can capture dietary characteristics not readily apparent from absolute intake measures alone. These findings suggest that rice intake may reflect limited dietary diversity, potentially limiting the intake of various nutrients (protein, fiber, micronutrients) from other food groups. However, since this substitution pattern appeared in both diabetic and non-diabetic networks, it should be interpreted as a general characteristic of Korean dietary culture rather than a diabetes-specific risk factor [34].

Our findings further revealed that non-diabetic dietary structures were characterized by diverse, interconnected food combinations centered on cuttlefish, cheese and pizza/hamburgers, and mushrooms, whereas diabetic structures showed simplified structures dominated by refined carbohydrate-based foods such as breads/spreads, dumplings and *Tteokguk* (rice cake soup). Importantly, the concept of dietary diversity in this study reflects a structural diversity in food co-consumption patterns, which is conceptually distinct from conventional food group-based diversity measures. Although the emergence of cheese and pizza/hamburgers as central foods in the non-diabetic network was unexpected, their absolute intake levels were low and showed minimal change across follow-up visits (baseline: 0.026, the first follow-up: 0.027, the second follow-up: 0.030 servings/day). This indicates that their centrality reflects their structural positioning within the overall co-consumption network rather than individual food intake alone or food-specific increases in intake. Similar structural differences between disease and non-disease networks have been reported in other metabolic conditions, including dementia and non-alcoholic fatty liver disease, where control networks tend to display a greater dietary diversity than case networks [29]. However, the direction of network structural differences may vary by disease context. For example, in a dietary network study on breast cancer, the case network exhibited a broader structure than the control network [35], underscoring that network complexity should be interpreted in a disease-specific manner. In the context of T2D, our findings are consistent with established evidence showing that a greater dietary diversity is associated with lower risk [36], whereas refined grain-dominated dietary patterns are positively associated with incidence [37]. Taken together, these observations suggest that a greater structural dietary diversity may be associated with a lower risk of T2D, although further studies are needed to clarify the underlying mechanisms and causal pathways.

From an applied perspective, the D_CCN framework may provide a structural lens for interpreting dietary exposures at both individual and population levels, complementing conventional intake-based approaches in nutritional epidemiology and public health. Further research is warranted to determine how these structural insights can be translated into intervention strategies.

Several limitations should be considered when interpreting our findings. First, although the FFQ was interviewer-administered by trained professionals and has been validated in the Korean population, recall bias and misreporting may still have affected dietary estimates. Aggregating 106 food items into 45 food groups, guided by clustering analysis and expert knowledge [20], was necessary for interpretability but led to the loss of within-group variation and may have masked dietary nuances. To mitigate random errors, we used cumulative average intakes from repeated assessments, which provide more stable estimates of long-term dietary patterns than single-time-point measures. Second, the generalizability may be limited both geographically and temporally. Because our study included only Korean adults, the observed network structures may not generalize to populations with different dietary practices. The relative homogeneity of traditional Korean diets, centered on rice and vegetable side dishes [30], resulted in networks where most foods clustered into a single large component rather than separating into distinct healthy and unhealthy modules. Consequently, hypergeometric distribution-based module detection had limited utility. Nonetheless, our differential network approach successfully identified disease-specific dietary structures even within a homogeneous population. Because network methods are inherently population-specific, further studies in populations with more heterogeneous diets and in contemporary cohorts are needed to determine whether the structural roles of specific foods change as dietary environments evolve. Third, network construction is less flexible for multivariable confounder adjustment than regression-based approaches. We partially addressed this by using partial correlations adjusted for energy intake and sex during network construction, and by applying comprehensive covariate adjustments in the modified Poisson regression models used to estimate incidence rate ratios.

## 5. Conclusions

In conclusion, this study proposed and validated a differential co-consumption network (D_CCN) approach to identify disease-specific dietary structures associated with T2D risk. The non-diabetes network was characterized by diverse and interconnected food combinations, whereas the diabetes network exhibited simplified, refined grain-centered structures. D_CCN scores derived from CAVAS showed consistent associations with incident T2D risk in the independent HEXA cohort, demonstrating cross-cohort transferability and highlighting the potential of network-based approaches for capturing population-specific dietary risk profiles.

## Figures and Tables

**Table 1:** Baseline characteristics of CAVAS and HEXA cohort participants.

Characteristics *	CAVAS			HEXA		
	Total Participants (*n* = 16,665)	Individuals Who Remained Non-T2D	Individuals Who Developed T2D	Total Participants (*n* = 51,206)	Individuals Who Remained Non-T2D	Individuals Who Developed T2D
Incident type 2 diabetes, *n* (%)	953 (5.7)			2190 (4.3)		
Follow-up duration, years	5.8 ± 3.9			4.9 ± 1.8		
Total person-years	96,605			205,454		
Incidence rate, per 1000 person-years	9.9			8.7		
Age, y	58.2 ± 9.7	58.1 ± 9.8	59.4 ± 8.5	53.0 ± 7.9	52.9 ± 7.9	55.1 ± 7.8
Female, %	63.0	63.4	56.7	67.7	68.3	53.9
Higher education ^1^, %	28.3	28.6	24.7	70.0	70.4	62.0
Regular exercise ^2^, %	21.7	21.8	20.0	38.8	38.8	39.2
Current smoker, %	15.0	14.8	19.0	10.2	9.9	17.4
Alcohol consumption, ml/d	12.1 ± 34.8	12.1 ± 35.0	12.8 ± 32.5	8.2 ± 27.1	8.1 ± 26.9	12.5 ± 29.8
Body Mass Index, kg/m^2^	24.3 ± 3.1	24.2 ± 3.1	25.8 ± 3.3	23.7 ± 2.8	23.7 ± 2.8	25.6 ± 3.1
Cumulative average total energy intake, kcal/d	1561 ± 425	1559 ± 422	1585 ± 458	1677 ± 406	1676 ± 404	1706 ± 443

* All values are expressed as means ± SD for continuous variables or percentages for categorical variables. ^1^ Higher education level (≥12 years of education). ^2^ Regular exercise (≥3 times/week and ≥30 min/session).

**Table 2:** Characteristics of top 10 hub foods ranked by degree centrality in co-consumption networks, stratified in diabetes-specific and non-diabetes-specific network.

Foods * (Serving/Day)	Food Consumption					Network Centrality Indices				
	Mean Consumption	SD Consumption	Median Consumption	N Consumers	Consumer Percentage	Degree	Betweenness	Closeness	Eigenvector	Strength
**NonDiabetic_Network,** ***n*** **= 15,712**										
Total rice	2.8	0.7	3	12,066	76.8	24	0.156	2.742	0.953	6.42
Vegetable dishes	0.5	0.7	0.3	11,784	75.0	23	0.170	2.647	1	6.57
Mushrooms	0.1	0.2	0	11,791	75.0	20	0.061	2.475	0.964	5.40
Processed meat/seafood	0.1	0.1	0	8174	52.0	16	0.093	2.572	0.681	3.84
Non-native fruit	0.1	0.2	0	11,988	76.3	16	0.089	2.547	0.661	3.92
Sliced raw fish and eel	0	0.1	0	7861	50.0	15	0.035	2.423	0.747	3.88
Shellfish	0.1	0.2	0	7996	50.9	15	0.017	2.326	0.781	4.03
Cheese and pizza/hamburgers	0	0.1	0	3866	24.6	14	0.043	2.439	0.645	3.42
Starch	0	0.1	0	8211	52.3	14	0.090	2.489	0.622	3.32
Beef	0.1	0.2	0	12,049	76.7	14	0	2.411	0.727	3.59
**Diabetic_Network,** ***n*** **= 953**										
Total rice	2.9	0.7	3	721	75.7	25	0.163	2.751	1	6.44
Vegetable dishes	0.5	0.8	0.3	715	75.0	22	0.236	2.607	0.933	6.05
Processed meat/seafood	0.1	0.1	0	495	51.9	18	0.056	2.474	0.796	4.66
Mushrooms	0.1	0.2	0	492	51.6	16	0.047	2.265	0.829	4.32
Tofu/bean sprouts	0.4	0.5	0.3	715	75.0	14	0.063	2.254	0.549	3.56
Shellfish	0.1	0.2	0	483	50.7	14	0.022	2.341	0.689	3.54
Non-native fruit	0.1	0.2	0	490	51.4	14	0.075	2.308	0.617	3.47
Beef	0.1	0.2	0	716	75.1	13	0.027	2.391	0.679	3.35
Sliced raw fish and eel	0	0.1	0	478	50.2	13	0.044	2.292	0.6	3.29
Starch	0	0.1	0	490	51.4	12	0.042	2.291	0.57	2.88

* Values for mean, standard deviation and median consumption are expressed as servings/day. Total rice (tRice), vegetable dishes (VegDish), mushrooms (Mushroom), processed meat/seafood (ProcMeat/Sea), non-native fruit (ImpFruit), sliced raw fish and eel (special fish) (RawFish), shellfish seafood (Shellfish), cheese and pizza/hamburgers (CheeseFast), starch (Starch), beef (Beef), and tofu/bean sprouts (TofuBeanspr) were abbreviated for network visualization and analysis. Consumer numbers represent participants classified as consumers based on binarized food intake data (see Section 2). Complete data for all 45 food groups are provided in Supplementary Table S2.

**Table 3:** Top five foods with high centrality value in the diabetes-specific and non-diabetes-specific subnetworks.

Network ^1^	Foods ^2^	Integrated Centrality Values	Degree	Betweenness	Eigenvector	Closeness	Strength	Network Role ^3^
**Non-Diabetic-Specific**								
1	Cuttlefish (Cuttlefish)	3.510	6	0.173	1.000	1.968	1.393	Primary Hub
2	Cheese and Pizza/Hamburgers (Cheese/Fast)	3.423	5	0.377	0.754	2.169	1.067	Hub Food
3	Mushrooms (Mushroom)	3.323	5	0.260	0.870	2.021	1.166	Hub Food
4	Poultry (Poultry)	2.662	4	0.156	0.692	1.637	0.916	Bridge
5	Non-Native Fruit (ImpFruit)	2.536	4	0.294	0.184	1.770	0.832	Bridge
**Diabetic-** **Specific**								
1	Breads/Spreads (Bread)	4.073	5	0.122	1.000	1.442	1.146	Primary Hub
2	Dumplings and *Tteokguk* (DumplTeok)	3.319	4	0.151	0.428	1.815	0.852	Hub Food
3	Eggs (Egg)	2.496	2	0.119	0.594	1.612	0.463	Hub Food
4	Poultry (Poultry)	2.432	2	0.127	0.443	1.769	0.458	Connector
5	Salt-Fermented Food (SaltFood)	2.221	3	0.061	0.172	1.303	0.636	Connector

^1^ The diabetes-specific subnetwork comprised 29 nodes, 25 edges, and 5 disconnected components; the non-diabetes-specific subnetwork comprised 23 nodes, 27 edges, and 2 disconnected components. ^2^ Foods are ranked by integrated centrality values, calculated as the mean of five standardized centrality measures (z-score). Abbreviations in parentheses indicate node names used in the network figures. ^3^ Network roles were classified hierarchically using five centrality measures: primary hub (rank 1 with eigenvector centrality ≥ 0.8), hub food (ranks 2–3), bridge food (betweenness centrality ≥ 0.15), connector (degree ≥ 2), and peripheral food (degree < 2). Integrated weight represents the mean of five z-score standardized centrality scores.

**Table 4:** Incidence rate ratio (IRR) and 95% confidence intervals (CIs) of type 2 diabetes incidence by quartiles of differential co-consumption network scores in CAVAS cohort and external validation in HEXA cohort using CAVAS-derived network weights.

Model *	Differential Co-Consumption Network Scores									
	CAVAS				*p*_trend_^†^	HEXA				*p*_trend_^†^
	Q1	Q2	Q3	Q4		Q1	Q2	Q3	Q4	
Median of the differential score (min, max)	0.98 (−9.90, 3.04)	4.57 (3.04, 5.96)	7.32 (5.96, 8.91)	11.02 (8.91, 24.6)		−0.23 (−13.7, 1.90)	3.51 (1.90, 4.87)	6.19 (4.87, 7.64)	9.49 (7.64, 24.0)	
Cases/person years	190/22,787	201/23,926	231/24,566	331/25,327		429/2050	492/2047	546/2064	723/2075	
Age, sex-adjusted	1.00	0.99 (0.82–1.21)	1.10 (0.91–1.33)	1.50 (1.25–1.79)	<0.0001	1.00	1.15 (1.01–1.30)	1.27 (1.12–1.44)	1.63 (1.45–1.84)	<0.0001
Multivariable IRR	1.00	0.98 (0.81–1.20)	1.07 (0.88–1.30)	1.45 (1.21–1.74)	<0.0001	1.00	1.13 (0.99–1.28)	1.23 (1.08–1.40)	1.58 (1.40–1.78)	<0.0001
+Further adjusted for fasting blood glucose at baseline	1.00	0.98 (0.81–1.19)	1.05 (0.87–1.27)	1.39 (1.16–1.67)	0.0001	1.00	1.14 (1.00–1.29)	1.22 (1.08–1.38)	1.59 (1.42–1.79)	<0.0001

* Multivariable model was adjusted for age (years), sex, higher education level (≥12 years of education), regular exercise (≥3 times/week and ≥30 min/session), smoking status (never/former/current), alcohol consumption (ml/d), body mass index (kg/m^2^) and total energy intake (kcal/d). ^†^ *p* values for linear trend were obtained by assigningthe median value of each quartile and treating it as a continuous variable using a modified Poisson regression with a robust error estimator.

## Data Availability

The datasets presented in this article are not readily available because of the data protection regulations of the Korea Disease Control and Prevention Agency (KDCA). Requests to access the datasets should be directed to the KDCA (https://www.kdca.go.kr/) (accessed on 29 January 2026).

## Supplementary Materials

The following supporting information can be downloaded at: https://www.mdpi.com/article/10.3390/nu18030506/s1. Table S1: Description of 45 foods; Table S2: Descriptive statistics and network centrality measures of 45 food groups by diabetes development status in CAVAS cohort; Figure S1: Flowchart of study in KoGES_CAVAS cohort and KoGES_HEXA cohort; Figure S2: Co-consumption network analysis workflow; Figure S3: Distribution of energy- and sex-adjusted partial correlation coefficients between food groups in CAVAS.; Figure S4: Conceptual illustration of the differential co-consumption network.
