# Supplementary material for: Differential Network-Based Dietary Structure and Type 2 Diabetes Risk: A Prospective Cohort Study Using Food Co-Consumption Networks
Source: Nutrients. 2026 Feb 2;18(3):506. doi: 10.3390/nu18030506 (PMC12899873; doi:10.3390/nu18030506)
Supplement: Supplementary file 1 [file nutrients-18-00506-s001.zip › nutrients-4113280-supplementary.pdf]

Supplementary Figure S1. Flowchart of study in KoGES_CAVAS cohort and KoGES_HEXA cohort

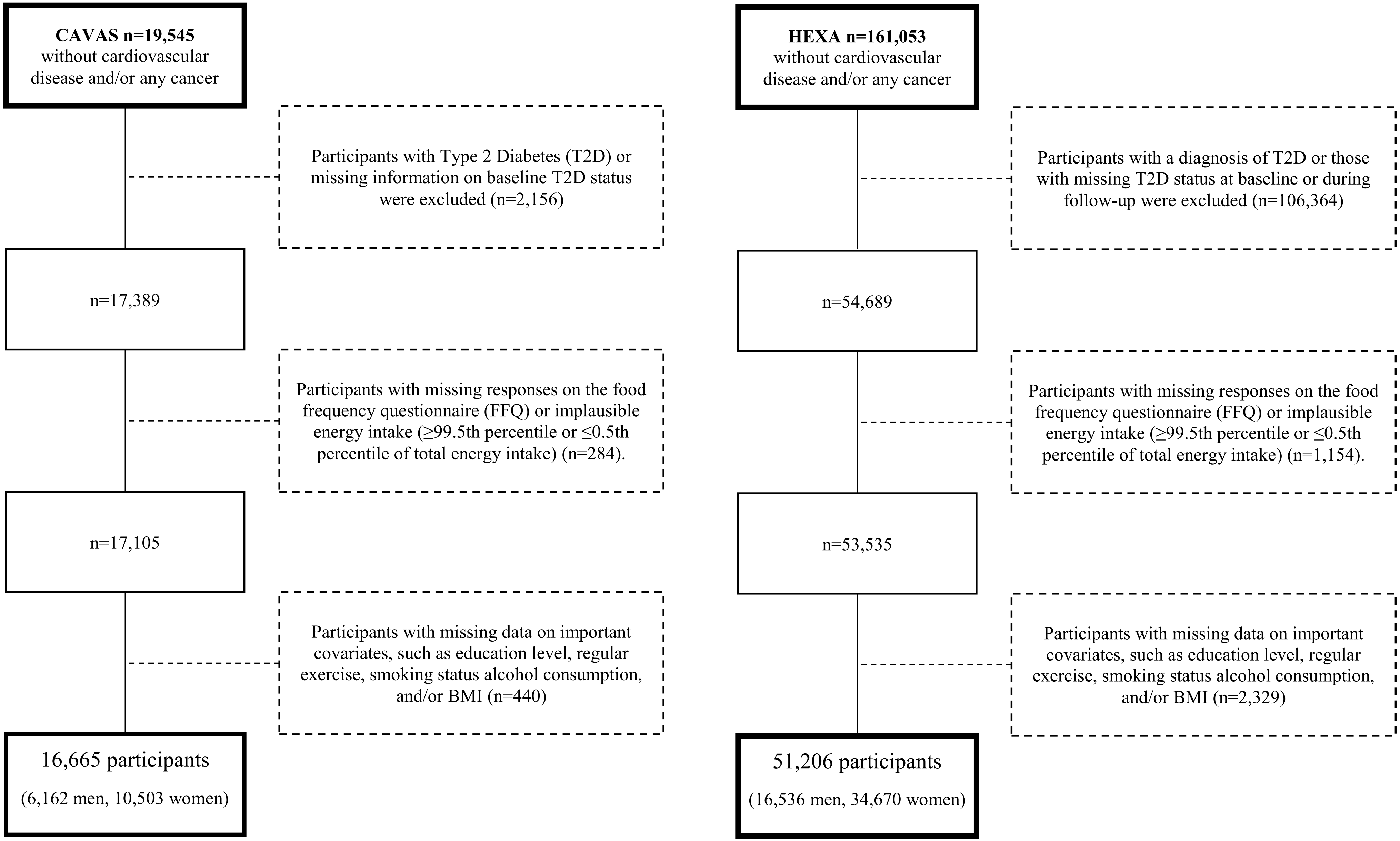

Supplementary Table 1. Description of 45foods

No.	Foods *(Serving/day)*	*Abbreviation*	No. of items*	Food items
1	Total rice*	*tRice*	5	Cooked white rice, Cooked rice with beans, Cooked rice with multi-grains
2	Noodles	*Noodles*	4	Ramyon, Kalguksu/Jangguk-noodles/Udon, Chajangmyon/ Champpong, Naengmyeon/Buckwheat noodles
3	Dumpling and Tteokguk	*DumplTeok*	2	Dumpling/Dumpling soup, plain stick shaped-rice cake/ Tteokguk (plain stick shaped-rice cake soup)
4	Rice cakes	*RiceCake*	1	Other rice cakes (Steamed rice cake, Baekseolgi, Injeolmi, etc.)
5	Cornflakes	*Cornflake*	1	Cornflakes
6	Breads/spreads	*Bread*	2	Loaf bread/sandwich/toast, jam/honey/margarine
7	Bread products	*SwBread*	3	Bread with small red bean, other breads (streusel bread/nut cake/castella/cream bread), cake /chocopie
8	Cheese & pizza/hamburger	*Cheese/Fast*	2	Pizza/hamburger, cheese
9	Grain powder	*GrainPowder*	1	Powdered meals/parched cereal powder
10	Snack/confections	*Snacks*	1	Cookie/cracker/snack, candy/chocolate
11	Nuts and roasted beans	*Nuts*	2	Peanut/almond/pine nut, soybeans/soybeans cooked in soy sauce (include green beans, exclude rice with beans)
12	Soybean paste soup	*SoySoup*	1	Soybean paste soup/fast-fermented bean paste/soybean paste/ Ssamjang
13	Tofu/bean sprouts	*TofuBeanspr*	2	Tofu (soft tofu, tofu stew, and tofu in tofu stew), bean sprouts/mung bean sprouts
14	Eggs	*Egg*	1	Egg/quail egg
15	Starch	*Starch*	2	Muk (starch jelly), Japchae (starch vermicelli)
16	Potatoes	*Potato*	2	Potatoes (steamed, French fries, soup, stew, jeon, etc.), sweet potatoes (steamed sweet potatoes, Mattang, etc.)
17	Baechukimchi	*BaechuKimchi*	1	Baechukimchi/Baegkimchi/Baechukimchi in Kimchi stew
18	Other kimchi	*OthKimchi*	3	Kkakdugi/Radish Kimchi, Nabakkimchi/Dongchimi, Other Kimchi (Pakimchi/Kodulbbagi/Gatkimchi)
19	Salt-fermented food	*SaltFood*	2	Other pickled vegetables (garlic pickles, garlic stems, radish pickles), salt-fermented fish (salted squid, salted intestine, salted pollack roe, salted shrimp, salted anchovies, salted clams, etc.)
20	Cabbage/radish Soup	*VegSoup*	2	Radish (soup, stew)/pickled radish, cabbage/cabbage soup
21	Vegetable dish	*VegDish*	7	Spinach (spinach namul, soup, etc.), vegetable wraps/vegetable salad (cabbage, lettuce, kale, chicory, bok choy, broccoli, etc.), other green vegetables (shepherd's purse, beetroot, curled mallow, mugwort, outer leaves, etc.), Doraji/Deoduck (kind of white root), bracken/sweet potato stems/taro stem, red pepper leaves/Chamnamul/Chwinamul, crown daisies/leek/water dropwort, carrot/carrot juice
22	Lettuce/perilla leaf	*Leaves*	2	Lettuce (Ssam (rice and condiments wrapped in leaves of lettuce)/salad, etc.), perilla leaf
23	Mushrooms	*Mushroom*	2	Oyster mushroom (Pleurotus ostreatus), Other mushrooms (Wood ear mushroom (Auricularia heimuer), Button mushroom (agaricus bisporus), Winter mushroom (flammulina velutipes) etc.))
24	Other vegetables	*OthVeg*	4	Cucumber, Onion, Green pepper, Pumpkin, immature
25	Sweet pumpkin	*SwPumpkin*	1	Pumpkin/Sweet pumpkin/Pumpkin juice
26	Poultry	*Poultry*	1	Fried chicken/Whole Chicken Soup/Samgyetang/Chicken Stew
27	By-products (organ meat, Seonji, Sundae)	*Bysprod*	1	By-products (Organ meat, Seonji, Sundae)
28	Cuttlefish	*Cuttlefish*	1	Cuttlefish/Dried cuttlefish/Small octopus
29	Pork	*Pork*	3	Pork belly, Pork-Pan roasted/Fried/Pork bulgogi/Meatball, Pork-steamed (Boiled pork, pork braised in soy sauce, pigs' feet)
30	Processed meat/Seafood	*ProcMeat/Sea*	3	Processed meat (Ham, sausage), Tuna, canned, Fish paste/Crab, flavored.
31	Beef	*Beef*	3	Steak/beef roast (Grilled ribs, Sirloin, Tenderloin, Beef bulgogi), Tang (Seolleongtang/Gomtang/Galbitang/ Doganitang), Soup (Beef soup, Yukgaejang, etc.)
32	Sliced raw fish and eel (special fish)	*RawFish*	2	Sliced Raw Fish, Eel
33	Dog meat	*DogMeat*	1	Dog meat
34	Fish	*Fish*	4	Blue-colored back fish (Mackerel/Pacific saury/Spanish mackerel), Hair tail, Yellow croaker/Snapper/Halibut, Alaska pollack/Frozen Alaska pollack/Dried Alaska pollock
35	Anchovy	*Anchovy*	1	Dried anchovy/Stir-fried dried anchovies
36	Seaweed	*Seaweed*	2	Laver-dried, Kelp/Sea mustard

37	Shellfish seafood	*Shellfish*	4	Clam (Small ark shell/Little neck clam/Clam meat)/Whelk (including Soup, Stew, Roast, Kalguksu, Salad, etc.), Oysters (including Salted oysters), Crab/Crab preserved in soy sauce, Shrimp
38	Milk/yogurt	*Milk*	2	Milk, Yogurt/Yoplait
39	Carbonated drink/ice cream	*Soda/Ice,*	2	Ice cream, Carbonated drinks (Coke, Sprite)
40	Soy milk	*SoyMilk*	1	Soy milk
41	Coffee	*Coffee*	3	Coffee, Coffee Sugar, Coffee Cream
42	Traditional beverages	*TradDrink*	1	Other beverages (Citron tea, Plum tea, Aloe, Persimmon punch, Ginseng tea, Sikhye, Jujube tea, Black herbal tea, etc.)
43	Green tea	*GTea*	1	Green tea
44	Native fruit[†]	*DomFruit*	10	Korean melon/Melon, Watermelon, Peach/Plum, Persimmon, hard/Persimmon, dried, Tangerine, Korean pear/Pear juice, Apple/Apple juice, Grapes/Grapes juice, Tomato/Tomato juice/Cherry tomato
45	Non-native fruit[‡]	*ImpFruit*	2	Banana, Orange/Orange juice

[*]The same validated 106-item food frequency questionnaire was administered at baseline and follow-up visits in both CAVAS and HEXA cohorts. We consolidated these food items by merging five rice-related items (cooked white rice, cooked rice with beans, cooked rice with multi-grains, and two other rice varieties) into a single rice category. We then applied Ward's hierarchical clustering method with the Silhouette method to determine optimal clusters, considering predefined cluster numbers (20, 30, 40). After examining cluster interpretability and consistency, we ultimately identified 45 modified food groups.

[†] Native fruit referred to fruits primarily grown and consumed in Korea.

[‡]Non-native fruit referred to fruits not traditionally cultivated in Korea but imported for consumption.

Supplementary Figure S2: Co-consumption Network Analysis Workflow

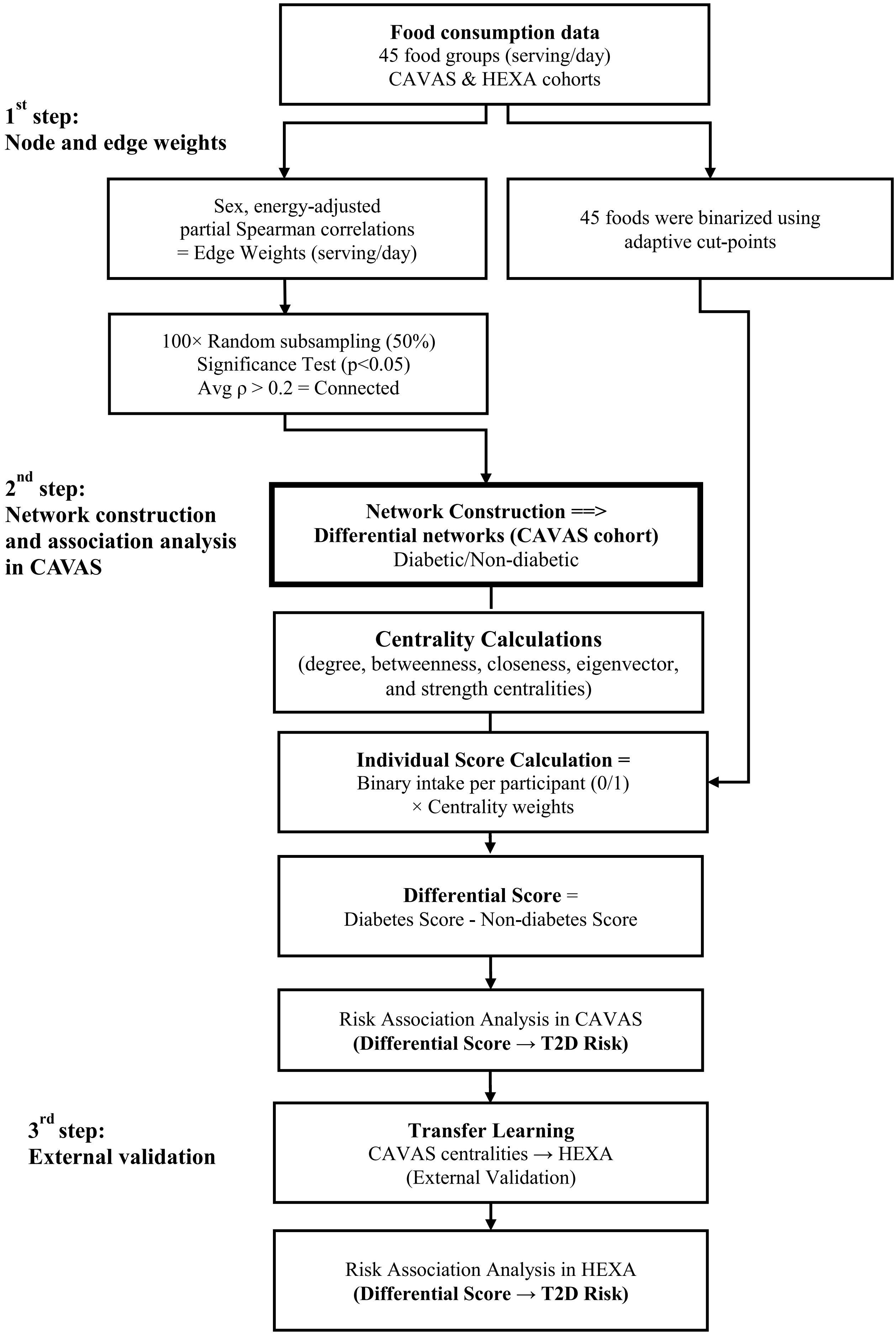

Supplementary Figure S3: Distribution of energy and sex-adjusted partial correlation coefficients between food groups in CAVAS

Red dashed line indicates the network inclusion threshold (r = 0.2), blue dashed line shows the third quartile (Q3 = 0.166). Both distributions demonstrate that the 0.2 threshold appropriately captures correlations above the upper quartile, ensuring meaningful co-consumption relationships while maintaining methodological consistency across datasets.

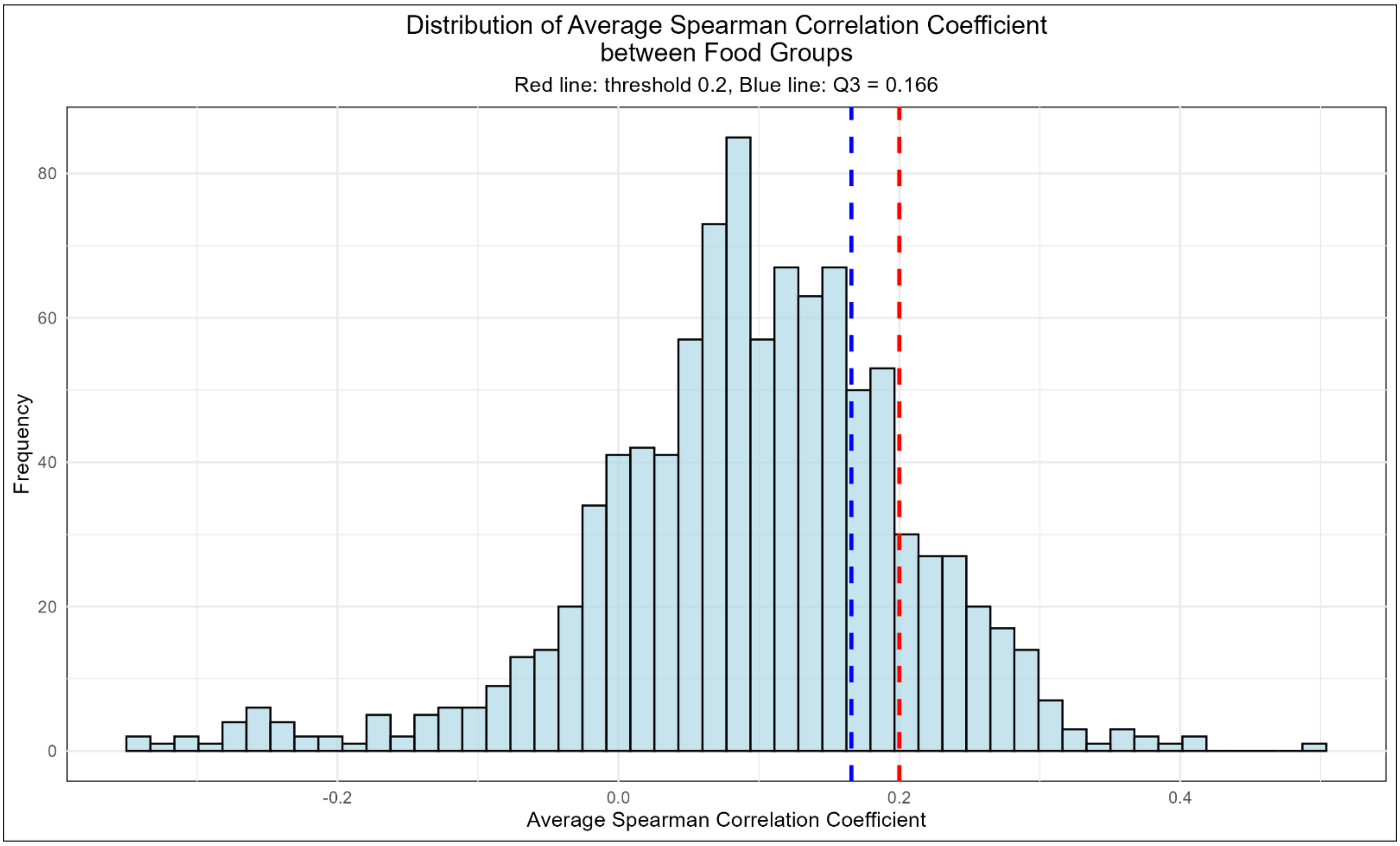

Supplementary Figure S4. Conceptual illustration of the differential co-consumption network

The differential co-consumption network (D_CCN) is constructed by extracting edges that appear exclusively in either the non-diabetic or diabetic network, adapted from the approach proposed by Naghizadeh et al. [14]. Shared edges (gray) reflect common dietary background and are removed, isolating disease-specific co-consumption relationships

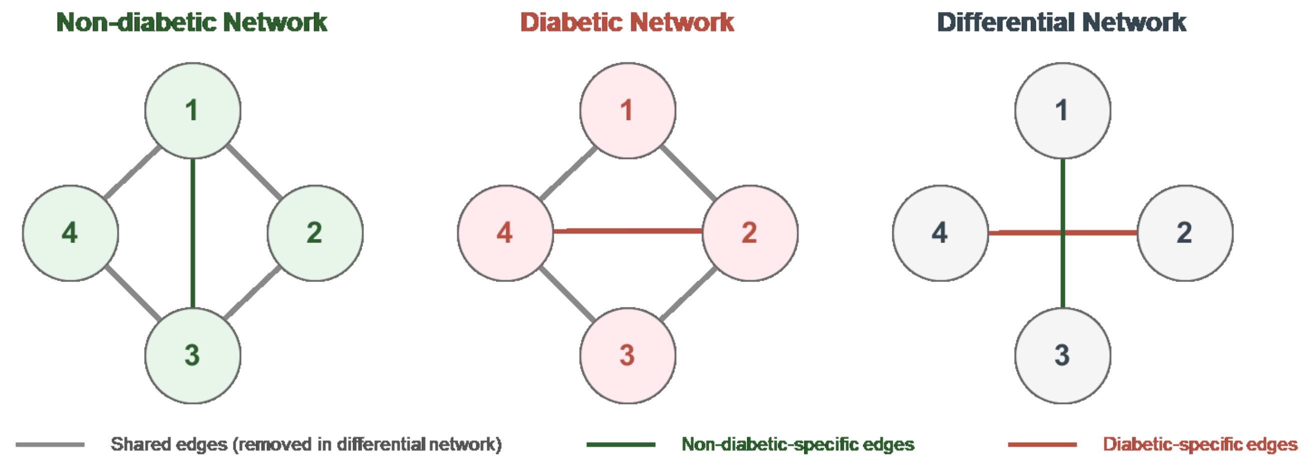

Supplementary Table 2. Descriptive statistics and network centrality measures of 45 food groups by diabetes development status in CAVAS cohort

Foods* (serving/day)	*Food Consumption Patterns*					*Network Centrality Indices*				
	**Mean consumption**	**SD consumption**	**Median consumption**	**N consumers**	**Consumer percentage**	**Degree**	**Betweenness**	**Closeness**	**Eigenvector**	**Strength**
NonDiabetic_Network (n=15,712)										
tRice	2.8	0.7	3	12,066	76.8	24	0.156	2.742	0.953	6.42
Noodles	0.2	0.2	0.1	11,859	75.5	2	0	1.652	0.08	0.53
DumplTeok	0	0.1	0	8,537	54.3	3	0.002	1.651	0.057	0.74
RiceCake	0	0.1	0	8,268	52.6	5	0.015	1.732	0.09	1.20
Cornflake	0	0	0	1,081	6.9	2	0	1.676	0.059	0.43
Bread	0.1	0.2	0	4,065	25.9	7	0.015	1.981	0.276	1.92
SwBread	0.1	0.2	0	8,205	52.2	8	0.017	2.079	0.249	2.21
Cheese/Fast	0	0.1	0	3,866	24.6	14	0.043	2.439	0.645	3.42
GrainPowder	0	0.1	0	4,114	26.2	0	0	0	0	0
Snacks	0.1	0.2	0	7,904	50.3	3	0.002	1.408	0.035	0.78
Nuts	0.2	0.3	0	7,930	50.5	8	0.033	2.165	0.372	1.91
SoySoup	0.5	0.5	0.3	11,789	75.0	5	0.002	1.633	0.096	1.14
TofuBeanspr	0.4	0.4	0.3	11,809	75.2	12	0.021	2.024	0.502	3.26
Egg	0.2	0.3	0.1	12,117	77.1	2	0	1.737	0.109	0.50
Starch	0	0.1	0	8,211	52.3	14	0.090	2.489	0.622	3.32
Potato	0.3	0.4	0.2	11,785	75.0	6	0.010	1.879	0.174	1.37
BaechuKimchi	2	1.1	1.8	11,809	75.2	1	0	1.169	0.007	0.31
OthKimchi	0.9	1.2	0.5	11,789	75.0	3	0.042	1.803	0.073	0.78
SaltFood	0.2	0.3	0.1	11,997	76.4	2	0	1.775	0.121	0.46
VegSoup	0.3	0.4	0.2	11,789	75.0	5	0.006	1.686	0.157	1.34
VegDish	0.5	0.7	0.3	11,784	75.0	23	0.170	2.647	1	6.57
Leaves	0.4	0.5	0.3	11,792	75.1	6	0.010	1.932	0.271	1.60
Mushroom	0.1	0.2	0	11,791	75.0	20	0.061	2.475	0.964	5.40
OthVeg	1.2	1.2	0.9	11,784	75.0	11	0.032	2.112	0.443	2.95
SwPumpkin	0	0.1	0	3,971	25.3	4	0	1.95	0.217	0.96
Poultry	0	0.1	0	8979	57.1	12	0.007	2.256	0.607	3.14
Bysprod	0	0	0	4911	31.3	8	0.013	1.896	0.315	1.85
Cuttlefish	0	0.1	0	8,505	54.1	12	0.013	2.309	0.592	2.91
Pork	0.1	0.2	0.1	11,867	75.5	7	0	1.954	0.371	1.92
ProcMeat/Sea	0.1	0.1	0	8,174	52.0	16	0.093	2.572	0.681	3.84
Beef	0.1	0.2	0	12,049	76.7	14	0	2.411	0.727	3.59
RawFish	0	0.1	0	7,861	50.0	15	0.035	2.423	0.747	3.88
DogMeat	0	0	0	4,015	25.6	2	0	1.584	0.074	0.45
Fish	0.3	0.4	0.2	11,816	75.2	12	0.021	2.166	0.645	3.24
Anchovy	0.3	0.4	0.2	11,867	75.5	6	0.005	1.716	0.173	1.50
Seaweed	0.5	0.5	0.4	11,787	75.0	9	0.004	2.072	0.416	2.34
Shellfish	0.1	0.2	0	7,996	50.9	15	0.017	2.326	0.781	4.03
Milk	0.5	0.6	0.3	11,883	75.6	2	0	1.645	0.149	0.61
Soda/Ice,	0.1	0.2	0	8,022	51.1	3	0.013	1.693	0.069	0.75
SoyMilk	0.1	0.2	0	3,961	25.2	0	0	0	0	0
Coffee	3.3	3.2	3	11,956	76.1	0	0	0	0	0

TradDrink	0.1	0.3	0	8,216	52.3	5	0	1.977	0.248	1.16
GTea	0.3	0.7	0	7,946	50.6	5	0.004	2.109	0.252	1.12
DomFruit	1.1	1	0.9	11,784	75.0	5	0	1.974	0.274	1.31
ImpFruit	0.1	0.2	0	11,988	76.3	16	0.089	2.547	0.661	3.92
Diabetic_Network (n=953)										
tRice	2.9	0.7	3	721	75.7	25	0.163	2.751	1	6.44
Noodles	0.2	0.2	0.1	715	75.0	2	0	1.668	0.105	0.53
DumplTeok	0	0.1	0	479	50.3	7	0.015	2.039	0.248	1.63
RiceCake	0	0.1	0	488	51.2	4	0.001	1.689	0.129	0.95
Cornflake	0	0	0	37	3.9	0	0	0	0	0
Bread	0	0.2	0	247	25.9	11	0.060	2.097	0.491	2.90
SwBread	0.1	0.1	0	512	53.7	9	0.011	1.984	0.35	2.43
Cheese/Fast	0	0.1	0	148	15.5	9	0.005	2.093	0.452	2.18
GrainPowder	0	0.1	0	239	25.1	1	0	1.256	0.005	0.22
Snacks	0.1	0.2	0	524	55.0	3	0	1.384	0.098	0.89
Nuts	0.2	0.3	0	477	50.1	6	0.025	2.144	0.276	1.36
SoySoup	0.5	0.5	0.4	716	75.1	7	0.007	1.903	0.229	1.76
TofuBeanspr	0.4	0.5	0.3	715	75.0	14	0.063	2.254	0.549	3.56
Egg	0.2	0.2	0.1	720	75.6	4	0.007	1.988	0.183	0.91
Starch	0	0.1	0	490	51.4	12	0.042	2.291	0.57	2.88
Potato	0.3	0.4	0.2	717	75.2	7	0.004	1.919	0.221	1.57
BaechuKimchi	2	1.2	2	721	75.7	1	0	1.095	0.01	0.34
OthKimchi	1	1.4	0.5	719	75.4	3	0.043	1.721	0.087	0.82
SaltFood	0.2	0.4	0.1	721	75.7	5	0.018	2.148	0.258	1.10
VegSoup	0.3	0.4	0.2	727	76.3	6	0.004	1.745	0.206	1.56
VegDish	0.5	0.8	0.3	715	75.0	22	0.236	2.607	0.933	6.05
Leaves	0.4	0.5	0.3	715	75.0	7	0.007	1.762	0.265	1.81
Mushroom	0.1	0.2	0	492	51.6	16	0.047	2.265	0.829	4.32
OthVeg	1.3	1.2	1	715	75.0	11	0.006	2.112	0.495	3.01
SwPumpkin	0	0.2	0	241	25.3	3	0	1.858	0.193	0.73
Poultry	0	0	0	516	54.1	10	0.008	2.023	0.445	2.58
Bysprod	0	0.1	0	310	32.5	9	0.017	1.959	0.326	2.12
Cuttlefish	0	0.1	0	498	52.3	6	0.007	1.918	0.281	1.43
Pork	0.1	0.2	0.1	719	75.4	6	0	1.845	0.341	1.65
ProcMeat/Sea	0.1	0.1	0	495	51.9	18	0.056	2.474	0.796	4.66
Beef	0.1	0.2	0	716	75.1	13	0.027	2.391	0.679	3.35
RawFish	0	0.1	0	478	50.2	13	0.044	2.292	0.6	3.29
DogMeat	0	0.2	0	248	26.0	2	0	1.521	0.074	0.48
Fish	0.3	0.5	0.2	715	75.0	11	0.034	2.212	0.538	2.77
Anchovy	0.3	0.4	0.2	715	75.0	7	0.004	1.689	0.218	1.79
Seaweed	0.5	0.6	0.3	715	75.0	8	0	2.135	0.408	2.09
Shellfish	0.1	0.2	0	483	50.7	14	0.022	2.341	0.689	3.54
Milk	0.5	0.6	0.2	728	76.4	2	0	1.626	0.164	0.59
Soda/Ice,	0.1	0.3	0	518	54.4	4	0	1.675	0.162	1.06
SoyMilk	0.1	0.2	0	259	27.2	2	0.043	1.734	0.064	0.43
Coffee	3.3	3.3	3	715	75.0	0	0	0	0	0
TradDrink	0.1	0.3	0	484	50.8	5	0	1.922	0.263	1.21
GTea	0.3	0.8	0	479	50.3	5	0.002	2.040	0.265	1.22
DomFruit	1.1	0.9	0.9	715	75.0	6	0.003	1.820	0.312	1.67

ImpFruit	0.1	0.2	0	490	51.4	14	0.075	2.308	0.617	3.47

* Values for mean, standard deviation and median consumption are expressed as servings/day. Consumer numbers represent participants classified as consumers based on binarized food intake data (see Methods). Network centrality indices are dimensionless measures derived from co-consumption correlation networks.
